# Renal pericytes: regulators of medullary blood flow

**DOI:** 10.1111/apha.12026

**Published:** 2012-11-06

**Authors:** T M Kennedy-Lydon, C Crawford, S S P Wildman, C M Peppiatt-Wildman

**Affiliations:** 1 Urinary System Physiology Unit, Royal Veterinary CollegeLondon, UK; 2 Medway School of Pharmacy, The Universities of Kent and Greenwich at MedwayKent, UK

**Keywords:** blood flow, cross-talk, medulla, pericyte

## Abstract

Regulation of medullary blood flow (MBF) is essential in maintaining normal kidney function. Blood flow to the medulla is supplied by the descending vasa recta (DVR), which arise from the efferent arterioles of juxtamedullary glomeruli. DVR are composed of a continuous endothelium, intercalated with smooth muscle-like cells called pericytes. Pericytes have been shown to alter the diameter of isolated and *in situ* DVR in response to vasoactive stimuli that are transmitted via a network of autocrine and paracrine signalling pathways. Vasoactive stimuli can be released by neighbouring tubular epithelial, endothelial, red blood cells and neuronal cells in response to changes in NaCl transport and oxygen tension. The experimentally described sensitivity of pericytes to these stimuli strongly suggests their leading role in the phenomenon of MBF autoregulation. Because the debate on autoregulation of MBF fervently continues, we discuss the evidence favouring a physiological role for pericytes in the regulation of MBF and describe their potential role in tubulo-vascular cross-talk in this region of the kidney. Our review also considers current methods used to explore pericyte activity and function in the renal medulla.

Renal blood flow is regionally specific, with cortical blood flow being tightly regulated by well-documented mechanisms (Kriz 1981, Pallone *et al*. 2003). Conversely, medullary blood flow (MBF) regulation is less well understood and remains highly controversial (Pallone *et al*. 1998, 2003). There is contradictory evidence regarding whether or not MBF is autoregulated, and numerous studies have focused on delineating the potential mechanisms that might be involved in this process (Pallone 1994, Pallone *et al*. 1998, Crawford *et al*. 2012). Our own findings on regulation of MBF have arisen from studies utilizing the *in situ* live kidney slice model, developed in our laboratory, which uniquely permits access to the intact inner and outer medullary regions of the kidney, and facilitates the examination of both vascular and tubular structures and cell function in parallel (Pallone 1994, Crawford *et al*. 2011). The application of this novel live slice model has facilitated detailed investigations in to the potential mechanisms underlying MBF regulation and tubulo-vascular cross-talk to be performed and has enabled us to characterize the key role that pericytes play in this phenomenon. We have demonstrated that *in situ* pericytes can respond to a variety of endogenous stimuli and in so doing play an important role in regulating vasa recta diameter. In this review, we discuss our experimental findings on the physiological role(s) pericytes may play in regulating MBF in the context of the wider literature in the field.

To help contextualize the role pericytes play in physiological regulation of MBF, we begin by (i) describing the structure of the medullary vasculature and how this lends itself to regulation of MBF, and (ii) describing the role pericytes play in regulating medullary microvessels and discuss the significance of tightly regulated MBF.

## Physiological regulation of medullary blood flow

Blood flow to the renal medulla is via vasa recta capillaries, which branch from efferent arterioles at juxtamedullary glomeruli, and run in parallel to the loops of Henlé and collecting ducts (Pallone *et al*. 2003). A second peritubular capillary bed, which branches from the efferent arterioles at cortical nephrons, surrounds the tubular structures in the cortex. Renal blood flow is constant, despite variations in arterial perfusion pressure. Changes in mean arterial blood pressure within the autoregulatory range (i.e. 90–200 mmHg) have little effect on renal blood flow or glomerular filtration rate. Renal blood flow is also regionally specific, with blood flow in the cortex tightly regulated. Experiments using the isolated *in vitro* blood-perfused juxtamedullary nephron technique have demonstrated autoregulation of afferent arteriolar blood flow (Takenaka *et al*. 1994, Harrison-Bernard & Navar 1996). Regulation of medullary blood flow is less well understood and initially thought to be passively regulated; more recent studies suggest renal medullary blood flow can be autoregulated independently of total renal blood flow (O'Connor *et al*. 2006, Rajapakse & Mattson 2011, Ahmeda & Johns 2012). Medullary blood flow regulation is important, because it must satisfy the conflicting demands of preserving the cortico-medullary gradients of NaCl and urea, while maintaining adequate oxygen and nutrient delivery, as well as metabolic clearance in the medulla. The vasa recta form capillary bundles in the outer and inner stripes of the outer medulla, entering as the descending vasa recta (DVR) that penetrate deep into the medulla before forming the ascending vasa recta (AVR). DVR are composed of a continuous endothelium, with pericytes juxtaposed at regular intervals along the microvessel. As the DVR advance to the inner medulla, pericytes become less frequent and diminish nearer the tip of the papilla (Sims 1986). Beyond this point, the endothelium becomes discontinuous, giving rise to the ascending vasa recta (AVR), which are characterized by their fenestrated endothelium (Kriz 1981, Pallone *et al*. 1998, 2003).

The parallel arrangement of descending and ascending limbs allows the vasa recta capillaries to form a countercurrent exchange system that maintains the cortico-medullary osmotic gradient established from countercurrent multiplication by the loops of Henlé, which is crucial for urine concentration (Jamison & Kriz 1982, Michel 1995, Cowley 1997, Pallone *et al*. 1998). Moreover, this parallel arrangement has a key role in regulating regional perfusion between the outer versus inner medulla (Cowley 1997). Contraction of the DVR results in the redirection of blood to the outer medullary inter-bundle capillaries and *vice versa* on dilation (Pallone *et al*. 1998). A consequence of this structural arrangement is low oxygen tension in the medulla: medullary partial pressure of oxygen is reported to be between 10 and 20 mmHg compared with 50 mmHg in the cortex (Brezis *et al*. 1991, Rosen *et al*. 1992, Liss *et al*. 1998). This creates a relatively hypoxic environment, (Eckardt *et al*. 2005), and failure to regulate it may result in severe hypoxia and ischaemic injury.

Medullary blood flow is <10% of total renal blood flow (RBF), and so tight regulation of the medullary microcirculation is essential for the preservation of normal kidney function. An increase in net MBF would result in ‘washout’ of the cortico-medullary osmotic gradient (Cowley 1997) and impaired urinary concentrating ability, whereas a sustained decrease in MBF would inevitably lead to ischaemia, which, depending on the severity and duration, could result in tissue (papillary) necrosis, scarring and chronic kidney injury (Fine *et al*. 2000, Nangaku 2006, Norman 2006).

It has been suggested previously that pericyte-mediated changes in DVR diameter can regulate MBF and alter its distribution in response to changes in active NaCl transport and the oxygen demands of renal tubular cells (Pallone 1994, Pallone & Mattson 2002, Crawford *et al*. 2011, 2012). Pericyte-mediated regulation of capillary diameter is achieved by pericyte contraction and relaxation, and it has also been demonstrated in other tissues, such as the central nervous system (CNS), with a similar functional significance attributed to pericytes (Rucker *et al*. 2000, Wu *et al*. 2003, Peppiatt *et al*. 2006).

## Experimental investigations of medullary blood flow

To date, the notion that MBF is regulated independently of arterial perfusion pressure remains controversial, despite the many experimental studies that have been undertaken to examine this concept. Several different experimental approaches have been adopted in various experimental animal models, and species and inter-strain differences have been reported.

Studies utilizing imaging techniques based on the dual-slit method [measurement of red blood cell (RBC) velocity at two separate points on the same capillary], to determine blood flow, velocity and vasa recta diameter in the exposed rat papilla concluded that MBF is regulated within a limited arterial pressure range (125–130 mmHg) and that regulation of MBF is governed upstream by renal arterial pressure (Cohen *et al*. 1983). Imaging studies focusing specifically on juxtamedullary blood flow similarly conclude that renal sensitivity to endogenous vasoactive mediators is confined to afferent and efferent arterioles and that downstream vasa recta were insensitive (Harrison-Bernard & Carmines 1994, 1995).

Conversely, when the same technique was used to investigate autoregulation of blood flow in the DVR of anti-diuretic rats, regulation of MBF was observed over a broad range of perfusion pressures (Cupples & Marsh 1988). Subsequent studies performed in alternative rat strains also reported regulated MBF, albeit over varying perfusion pressure ranges: 101–132 mmHg in Munich Wistar rats compared with 104–126 mmHg in spontaneously hypertensive rats, and 90–114 mmHg in Sprague Dawley rats (Farrugia *et al*. 1992, Larson & Lockhart 1995). The inability to effectively assess autoregulation in these studies was attributed to animals being exposed to varying and inconsistent ranges in renal perfusion pressures (Cupples & Braam 2007). Studies that have utilized *in vivo* laser Doppler flowmetry (LDF) techniques to measure regional red blood cell flux (in rats, rabbits and dogs) have also failed to provide any clear answers (Stern *et al*. 1979, Takezawa *et al*. 1987, Roman *et al*. 1988, Mattson *et al*. 1993, Huang *et al*. 1994, Strick *et al*. 1994, Lerman *et al*. 1995, Harrison-Bernard & Navar 1996, Majid & Navar 1996, Majid *et al*. 1997, 1998, 1999, 2001, Nafz *et al*. 1998), perhaps due in part to the inconsistent perfusion pressure ranges applied in each study (Roman *et al*. 1988, Huang *et al*. 1994). LDF studies carried out on larger mammals have provided more consistent evidence favouring regulated MBF (Majid *et al*. 1997, Eppel *et al*. 2004, Cupples & Braam 2007). Studies performed in rabbits reported a constant MBF, despite increasing renal arterial pressure (Eppel *et al*. 2003).

Collectively, these studies not only serve to emphasize the potential for species and inter-strain differences in regulation of MBF, but also reveal limitations of the different techniques used. Erythrocyte velocity for example is thought to be less well regulated at the papilla than in the medulla and cortex (Cohen *et al*. 1983, Roman *et al*. 1988, Farrugia *et al*. 1992), and papillary autoregulation is deemed to be highly sensitive to changes in volume expansion (Roman *et al*. 1988); thus, imaging experiments performed on the exposed papilla must be interpreted with caution. LDF studies rely on accurate probe calibration and precise placement of probes in the renal parenchyma, which is difficult especially in smaller animals; moreover, kidney size may vary between animals further hampering consistent localization of probes in the renal parenchyma. Lastly, it is possible that the experimental discrepancies reported regarding regulation of MBF could arise from differential regulation of blood flow in the outer and inner medulla, as proposed in Pallone's hypothesis of redistribution of flow in the medulla (Pallone & Silldorff 2001). If blood flow is redistributed from the papilla towards the outer medulla, this would explain the apparent decrease in autoregulatory efficiency observed in the papilla. While redistribution of medullary blood flow has yet to be demonstrated experimentally, it provides a tantalizing explanation for the conflicting data on autoregulation of MBF and merits further investigation.

## Renal pericytes

Despite the emerging evidence in favour of regulated MBF (Cupples & Marsh 1988, Pallone 1994, Pallone & Silldorff 2001, Crawford *et al*. 2011, 2012) and evidence describing regulated capillary blood flow in other organs and tissues (Krogh 1929, Wu *et al*. 2003, Peppiatt *et al*. 2006), the dogma that capillary blood flow is passive and driven by upstream arteriolar control of perfusion pressure has been a widely accepted mechanism until recently. Historically, this assumption was based on the anatomical observation that arteries and arterioles are encircled by contractile smooth muscle cells, critical for vasoconstriction and vasodilation, and that capillaries apparently are not. The presence of pericytes along microvessels and their contractile capabilities was ‘however’ revealed in the late 19th and early 20th centuries (Rouget 1879, Krogh 1929), although this was largely overlooked until more recently (Pallone 1994, Pallone & Silldorff 2001, Pallone & Mattson 2002, Kawamura *et al*. 2004, Peppiatt *et al*. 2006, Puro 2007, Crawford *et al*. 2011, 2012).

**Figure 1:**
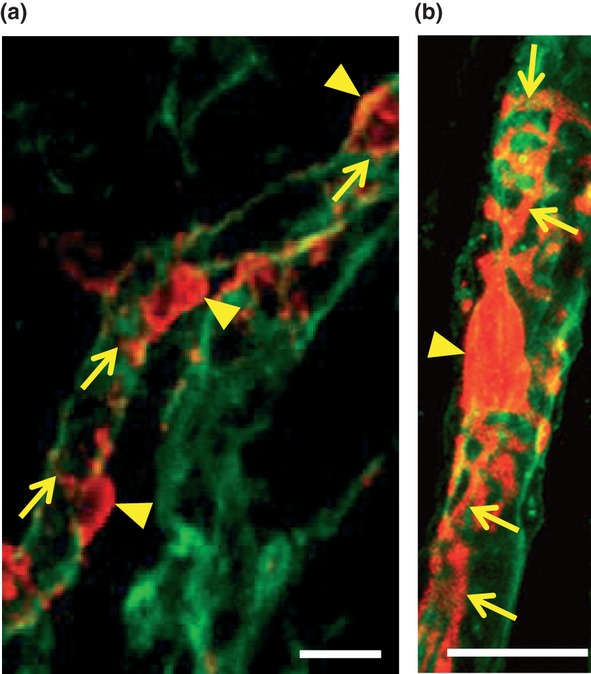
Identification of pericytes in the renal medulla. Kidney tissue slices (200 μm, from adult, male Sprague Dawley rats) were labelled with Alexa-488-conjugated IB4 and anti-NG2 (probed with Alexa 555 secondary antibody) to identify vasa recta capillaries and pericytes respectively. Pericytes (red) are identified on vasa recta capillaries (green; a). Pericyte cell bodies (arrowheads; a) are located on the abluminal side of the capillary. Finger-like processes (arrows; a) extend from the cell body to run along and wrap around the capillary [image adapted from (Crawford *et al*. 2012)]. Kidney slices (200 μm) obtained from NG2 Ds-Red Bac transgenic mice, in which pericytes express fluorescent Ds-Red (red; b) and vasa recta capillaries were labelled with Alexa-488-conjugated IB4 (green; b). Processes (arrows; b) are shown extend from the cell body (arrowhead; B). Primary processes are seen to extend along the length of the capillary, and secondary processes wrap around the vessel (Crawford C and Peppiatt-Wildman CM, unpublished data). Scale bar = 10 μm.

Pericytes are singular smooth muscle-like cells residing on the abluminal side of the endothelium. As their name implies, pericytes are perivascular cells consisting of a cell body with numerous claw-like processes that branch from the cell body and ‘wrap around’ microvessels, including arterioles, capillaries and venules (Shepro & Morel 1993, Hirschi & D'Amore 1996, Bergers & Song 2005). NG2 is an extracellular proteoglycan expressed by pericytes, and the anti-NG2 antibody has been used to identify pericytes in many organs, including vasa recta capillaries of the kidney, (Ozerdem *et al*. 2001, Peppiatt *et al*. 2006, Virgintino *et al*. 2007, Huang *et al*. 2010, Crawford *et al*. 2011, 2012, Mogensen *et al*. 2011), revealing a complex network of processes that extend from the cell body along the axis of, and wrap around, the capillary (Fig. 1a). In NG2-DsRed BAC transgenic mice, cells that express NG2 fluoresce red (Zhu *et al*. 2008, Hamilton *et al*. 2010), thus facilitating the identification of pericytes throughout the whole organism, including the kidney (Fig. 1b). Pericytes have been identified in all organs and tissues in a wide range of species [for review see (Sims 1986)] and have a variety of contrasting functional roles such as vessel stabilization, endothelial cell regulation, angiogenesis and phagocytosis. Pericytes have also been described as mesenchymal stem cells, because of their potential to differentiate into different cell types (Dore-Duffy *et al*. 2006, Crisan *et al*. 2011, Montemurro *et al*. 2011). Of particular interest in this review is the role of pericytes in the regulation of capillary blood flow (Sims 1986, Shepro & Morel 1993, Hirschi & D'Amore 1996, Bergers & Song 2005). Pericytes typically express smooth muscle α-actin (α-SMA), which provides the contractile machinery necessary for the regulation of vessel diameter, and it has been shown to be expressed by renal pericytes (Park *et al*. 1997).

The precise location of pericytes along a vessel wall is thought to be determined by the functional demand of the tissue being serviced (Sims 2000, Bergers & Song 2005). The density of pericytes in different tissues is known to vary, pericyte density being greater in the kidney than in many other tissues, such as the CNS and retina (Sims 1986, Bergers & Song 2005, Crawford *et al*. 2012). Within the kidney, pericyte density also varies, the density being greater in the outer medulla compared with the inner medulla (Crawford *et al*. 2012). In fitting with our findings regarding pericyte density in the renal medulla, it has previously been reported that pericyte density in the kidney increases to meet the metabolic demands of the tissue region in which they reside (Park *et al*. 1997).

Given the complexity of MBF and its role in facilitating urine concentration, the need for tight local control of blood flow, independent of changes in cortical blood flow, is perhaps not surprising. Regulation of vasa recta blood flow and the role of pericytes in regulating vasa recta capillary diameter have been investigated extensively by Pallone and colleagues who utilized the isolated perfused vasa recta model. This experimental model proved to be pivotal in identifying which agents were mediating vasa recta constriction/dilation and in attributing a functional role to contractile pericytes. Vasa recta pericytes were shown to evoke both vasoconstriction and dilation of isolated perfused DVR in response to a number of different agonists, many of which are endogenous to the medulla: angiotensin-II (Ang-II), endothelin-1 (ET-1), nitric oxide (NO), adenosine and prostaglandin E_2_ (PGE_2_) (Pallone 1994, Pallone & Silldorff 2001).

## Endogenous vasoactive agents and their effect on renal pericytes

**Figure 2:**
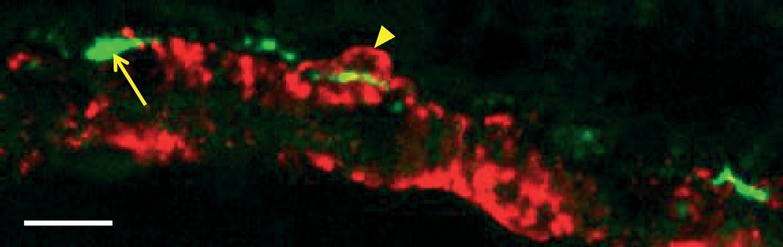
Co-localization of pericytes and sympathetic nerves in the renal medulla. Kidney tissue slices (200 μm, from adult, male Sprague Dawley rats) were labelled with anti-NG2 (probed with Alexa 555 secondary antibody) to identify vasa recta pericytes (red, arrowhead). Sympathetic nerve varicosities were identified with an anti-tyrosine hydroxylase antibody, amplified with a biotinylated secondary antibody that was probed with FITC-conjugated tertiary antibody (green, arrow). Confocal image shows pericytes (red) on a vasa recta capillary, co-localized and in close proximity to sympathetic nerves (green) (Crawford *et al*. 2012). Scale bar = 10 μm.

There are many vasoactive agents endogenous to the kidney that might physiologically regulate MBF. The pericyte-mediated vascular responses to these agents are summarized in Tables 1 and 2. Both vasa recta endothelial cells and tubular epithelial cells are established sources of medullary vasoactive signals [ET-1, Ang-II, PGE_2_, NO, adenosine, adenosine triphosphate (ATP),] as are interstitial cells (PGE_2_) and sympathetic nerves [noradrenaline (NA), ATP] (de Nucci *et al*. 1988, Pallone 1994, Silldorff *et al*. 1995, 1996, Yang *et al*. 1995, Pallone & Mattson 2002, Peppiatt *et al*. 2006, Crawford *et al*. 2011, Edwards *et al*. 2011). Anatomical studies have identified sympathetic nerves in the renal medulla (Eppel *et al*. 2004), predominantly in the outer medullary DVR (OMDVR) (Yang *et al*. 1995), and we have elaborated on these studies by specifically identifying sympathetic nerves in close apposition to pericytes in the outer medulla [Fig. 2 (Crawford *et al*. 2012)]. Given that pericytes are known to respond to acetylcholine (ACh), NA and ATP (Eglen *et al*. 1994, Yang *et al*. 1995, Crawford *et al*. 2011, 2012), and their close proximity to sympathetic nerve terminals, it is likely that neuronal release of NA and ATP contributes to the regulation of MBF.

**Table 1:** Endogenous stimuli that evoke a pericyte-mediated vasoconstriction of vasa recta

Stimulus	Source of vasoactive agent	Receptor activated	Receptor location	References
Acetylcholine	Parasympathetic nerves	Muscarinic	Functional evidence for Mus Rs on pericytes, no gene expression studies	Eglen *et al*. 1994, Yang *et al*. 1995,
Angiotensin-II	Endothelial cells	AT_1_	AT_1_: vasa recta bundles, interstitial cells and collecting duct epithelium	Mujais *et al*. 1986, Zhuo *et al*. 1992, Edwards & Aiyar 1993, Seldin & Geibisch 2008, Crawford *et al*. 2012,
Adenosine triphosphate (ATP)	Tubular epithelium, endothelial cells, RBCs	P2 receptors	DVR, loop of Henle and collecting duct epithelium	Sprague *et al*. 1996, Jans *et al*. 2002, Unwin *et al*. 2003, Praetorius *et al*. 2005, Wildman & King 2008, Crawford *et al*. 2011,
Endothelin-1	Endothelial cells and collecting duct epithelium	ET_A_	Collecting duct epithelium, vascular bundles, RMIC	de Nucci *et al*. 1988, Silldorff *et al*. 1995, Crawford *et al*. 2012,
Noradrenaline	Sympathetic nerves	α_1_-adreno-receptors	OM vasa recta	Yang *et al*. 1995, DiBona & Kopp 1997, Crawford *et al*. 2012,
UTP	Tubular epithelium, endothelium, plasma	P2 receptors	DVR, loop of Henle and collecting duct epithelium	Lazarowski & Boucher 2001, Jans *et al*. 2002, Unwin *et al*. 2003, Praetorius *et al*. 2005, Wildman & King 2008, Crawford *et al*. 2011,
Vasopressin	Hypothalamus (circulation)	V_1a_	V_1_: medullary vasculature, thin ascending limbs and OM collecting duct	Ostrowski *et al*. 1993, Nielsen *et al*. 1995, Turner & Pallone 1997, Cowley 2000

ATP, Adenosine triphosphate; RBC, Red blood cells; RMIC, renal medullary interstitial cells; UTP, uridine triphosphate.

**Table 2:** Endogenous stimuli that evoke a pericyte-mediated vasodilation of vasa recta

Stimulus	Source of vasoactive agent	Receptor activated	Receptor location	References
Acetylcholine (NO mediated)	Parasympathetic nerves	Muscarinic	Functional evidence for Mus Rs on pericytes	Eglen *et al*. 1994, Yang *et al*. 1995,
Adenosine	mTAL	A_1_ and A_2a_, A_2b_, sub-types	A_1_: DVR, loops of Henle and collecting duct epithelium.	Silldorff *et al*. 1996, Kreisberg *et al*. 1997, Guan *et al*. 2007, Crawford *et al*. 2011, Silldorff & Pallone 2001,
			A_2a_: collecting duct epithelium.	
			A_2b_: loops of Henle	
Angiotensin-II (NO mediated)	Endothelial cells	AT2 (dilation- NO mediated)	AT2: renal arteries (higher expression in foetal and neonatal renal tissues)	Zhuo *et al*. 1992, Edwards & Aiyar 1993, Pallone 1994, Seldin & Geibisch 2008, Crawford *et al*. 2012,
ATP (concentration dependent, NO mediated)	Tubular epithelium, endothelial cells, RBCs	P2 receptors	DVR, loop of Henle and collecting duct epithelium	Burnstock 1987, Sprague *et al*. 1996, Jans *et al*. 2002, Unwin *et al*. 2003, Praetorius *et al*. 2005, Wildman & King 2008, Erlinge & Burnstock 2008, Crawford *et al*. 2011,
Nitric Oxide	RBCs, endothelial cells, collecting duct epithelium	(Freely diffuses into cells)	–	Mattson & Higgins 1996, Wu *et al*. 1999, Dickhout *et al*. 2002, Cao *et al*. 2010, Edwards *et al*. 2011, Crawford *et al*. 2012,
PGE_2_	RMIC, collecting duct epithelium	EP2, EP4	EP2: descending thin loop and OM vasa recta.	Pallone 1994, Jensen *et al*. 2001, Crawford *et al*. 2012,
			EP4: Collecting duct epithelium, OM vasa recta	
Vasopressin	Hypothalamus (circulation)	V_2_	V_2_: Collecting Duct	Ostrowski *et al*. 1993, Nielsen *et al*. 1995, Turner & Pallone 1997, Cowley 2000

ATP, Adenosine triphosphate; DVR, Descending vasa recta; RBC, Red blood cells; RMIC, renal medullary interstitial cells.

In addition to peptide and hormonal vasoactive agents, free radicals such as NO and reactive oxygen species (ROS) are also known to be potent regulators of MBF (Cao *et al*. 2010, Edwards *et al*. 2011). The renal medulla is known to have a far greater capacity for NO production than the cortex (Biondi & Romero 1990, Moridani & Kline 1996, Mattson & Wu 2000), and NO has been intrinsically linked to changes in DVR lumen and solute transport along the nephron (Plato & Garvin 1999, Pallone & Mattson 2002). All three isoforms of nitric oxide synthase (NOS) – neuronal NOS (nNOS), inducible NOS (iNOS) and endothelial NOS (eNOS) – have been identified on vascular and tubular structures in the kidney (Bachmann & Mundel 1994, Mattson & Higgins 1996). NO bioavailability is also governed by reactive oxygen species (ROS). ROS are generated by one and two electron reductions of O_2_, resulting in the formation of either superoxide ion (O_2_^−^.), hydrogen peroxide (H_2_O_2_) or hydroxyl free radical (OH) (Fridovich 1995, Thannickal & Fanburg 2000). ROS favour vasoconstriction and are increasingly being associated with hypertension and diabetic nephropathy (Schnackenberg *et al*. 1998, Touyz 2003). ROS-evoked vasoconstriction is mediated following peroxynitrite formation in response to superoxide interacting with locally released NO. In comparison with NO, peroxynitrite evokes a weak vasodilation, thus resulting in a net reduction in MBF (Rubanyi & Vanhoutte 1986, Pallone & Mattson 2002). ROS-mediated vasoconstriction is known to be attenuated by antioxidants, such as superoxide dismutase (SOD), and by the SOD mimetic TEMPOL, both of which have been shown to increase MBF by as of yet undetermined mechanisms (Schnackenberg *et al*. 1998, Zou *et al*. 2001).

Functional studies using N5-[imino(nitroamino)methyl]-L-ornithine, methyl ester, monohydrochloride (L-NAME), a NO inhibitor, have demonstrated that inhibition of endogenous NO in live kidney slices shows pericyte-mediated vasoconstriction of vasa recta capillaries (Crawford *et al*. 2012), whereas the application of S-nitroso-N-acetylpenicillamine (SNAP), a NO donor, to live kidney slices evoked a pericyte-mediated vasodilation of *in situ* vasa recta (Crawford *et al*. 2012).

Vasoactive stimuli are released in the medulla by autocrine or paracrine mechanisms and act at their respective receptors expressed on DVR pericytes to mediate their effects. It has been suggested that feedback of vasoactive stimuli to juxtamedullary resistance vessels may provide the medulla with an intrinsic feedback loop, which could allow the medulla to control its own perfusion (Pallone & Silldorff 2001). In principle, this feedback system could operate on the basis that stimuli leaving the medulla via the AVR would be in close proximity to pericytes on the DVR at the outer medullary vascular bundles; thus, stimuli with a sufficiently long half-life could exert their vasoactive effects a second time. This concept is in keeping with the hypothesis of locally controlled MBF, and it merits further investigation.

## Tubulo-vascular cross-talk

**Figure 3:**
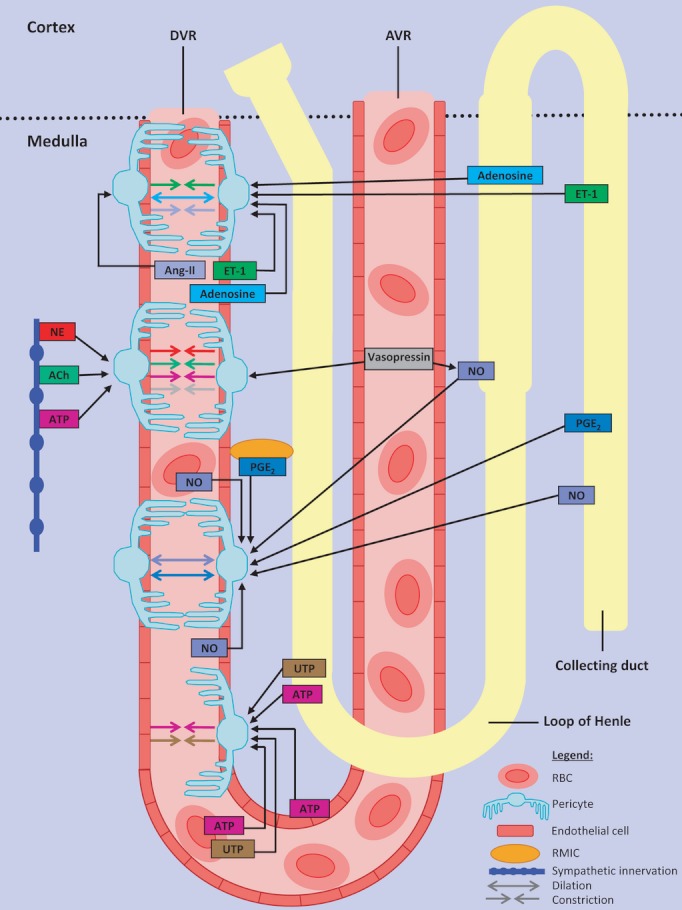
Proposed signalling mechanisms involved in pericyte-mediated regulation of descending vasa recta (DVR) diameter. Various endogenous vasoactive mediators are released from neighbouring tubular epithelium, vascular endothelium, red blood cells (RBC) and renal medullary interstitial cells (RMIC) of the renal medulla. These compounds signal to pericytes residing on DVR capillaries to cause pericyte-mediated vasoconstriction (arrows→←, endothelin-1 [ET-1], prostaglandin E_2_ [PGE_2_]_,_ nitric oxide [NO], adenosine, uridine triphosphate [UTP], adenosine triphosphate [ATP], acetylcholine [Ach] and circulating hormones: angiotensin [Ang-II], vasopressin, noradrenaline [NA]) or vasodilation (arrows ↔, NO and PGE_2_).

The close proximity of tubular and vascular structures in the medulla provides the ideal setting for tubulo-vascular cross-talk mechanisms to operate. We propose that pericyte cells bridge the gap between tubular and endothelial cell signalling mechanisms and that they detect vasoactive stimuli released from the epithelial, interstitial and endothelial cells and respond by regulating vasa recta diameter and consequently MBF (Crawford *et al*. 2011, 2012). The actual source of the vasoactive signal could be tubular, endothelial or neuronal, and both autocrine and paracrine pathways might be responsible for its release (Fig. 3).

Potential examples of pericyte-mediated tubulo-vascular cross-talk might be inferred from studies not primarily focused on investigating this mechanism directly. Here, we provide some examples that demonstrate the potential role of pericytes in reciprocally coordinating medullary nephron function and blood flow demands. In the medulla, for example, adenosine acts as a vasodilator via pericytes (Silldorff & Pallone 2001), therefore increasing MBF (Agmon *et al*. 1993). Medullary oxidative stress arising from a pathological insult can cause adenosine release from the medullary thick ascending limb (mTAL) (Beach & Good 1992), which can dilate neighbouring vasa recta by its action at pericytes (Crawford *et al*. 2011), thereby increasing oxygen supply and simultaneously reducing oxygen consumption by direct inhibition of NaCl reabsorption in the mTAL (Agmon *et al*. 1993, Zou *et al*. 1999, Pallone *et al*. 2003).

ET-1 constricts isolated and *in situ* DVR (Silldorff *et al*. 1995, Crawford *et al*. 2012) and inhibits absorption of sodium and water in the mTAL (Plato *et al*. 2000). ET-1 is synthesized by both collecting duct (CD) epithelial cells and endothelial cells, and once it is released it acts locally, unless it is taken up into the circulation (de Nucci *et al*. 1988). The mTAL, CD and vasa recta are all closely apposed; therefore, locally released ET-1 (epithelial or endothelial) would act via pericytes to reciprocally regulate vasa recta diameter (Crawford *et al*. 2011). We have demonstrated that Ang-II and NO exhibit their vasoactive effects specifically via pericytes (Crawford *et al*. 2012), and Ang-II-mediated constriction of DVR in medullary ray tissue strips is attenuated by NO released from neighbouring mTAL epithelium (Dickhout *et al*. 2002).

Similarly, the constrictive effects of both ET-1 and Ang-II are subject to attenuation by vasodilatory PGE_2_, which is released by renal medullary interstitial cells (RMIC) and collecting duct epithelial cells (Pallone 1994). The ensuing increase in blood flow brought about by PGE_2_-mediated vasodilation is thought to increase NaCl excretion (Silldorff *et al*. 1995), and PGE_2_ itself may modulate solute absorption along the nephron through its direct action at pericytes. Crawford *et al*. (2011) have demonstrated ATP- and uridine triphosphate (UTP)-mediated constriction and dilation of *in situ* vasa recta, specifically at pericyte sites (Crawford *et al*. 2011). The most likely sources of these extracellular nucleotides are endothelial cells, red blood cells, tubular epithelial cells and sympathetic nerves (Sprague *et al*. 1996, Jans *et al*. 2002, Praetorius *et al*. 2005). Most recently, it has been demonstrated that exposing live kidney slices to hypotonic insult, so as to release ATP, results in pericyte-mediated vasodilation of *in situ* vasa recta, which is direct evidence for tubulo-vascular cross-talk in the medulla (Crawford *et al*. 2012). Here, contractile pericytes act by sensing a change in the concentration of extracellular nucleotides and respond by relaying this to the vasculature in order to fine-tune MBF. In all examples described here, it appears that pericytes are acting as a biological transducer and are pivotal in ultimately determining vessel diameter and blood flow.

In support of this key role for pericytes in the kidney, there are similar studies describing pericyte communication or interactions with adjacent cell types in other systems. For example, retinal and cerebellar pericytes respond to changes in the metabolic demands of the surrounding neurones and alter capillary diameter accordingly; however, the exact mechanism by which this occurs is still unclear (Wu *et al*. 2003, Peppiatt & Attwell 2004, Peppiatt *et al*. 2006).

## Role of pericytes in pathophysiology: renal fibrosis

In the retina, it is well established that loss of pericyte function is an instrumental factor in the development of pathological conditions such as diabetic retinopathy (Sakagami *et al*. 1999, Cai & Boulton 2002, Tu *et al*. 2011). Indeed, pericyte dysfunction is implicated in other pathological conditions such as tumour angiogenesis and atherosclerosis (Benjamin *et al*. 1999, Morikawa *et al*. 2002, Yamagishi & Imaizumi 2005), and more recently in Alzheimer's disease (Bell *et al*. 2010).

In the kidney, a role for pericytes in the pathogenesis of renal fibrosis and progression of chronic kidney disease has been proposed (Lin *et al*. 2008, Duffield & Humphreys 2011, Schrimpf & Duffield 2011, Smith *et al*. 2012). It has been suggested that detachment and migration of pericytes from renal microvessels and their differentiation to myofibroblasts (defined as fibroblasts with contractile capabilities) cause vessel destabilization, capillary rarefaction and loss. Loss of vessels ultimately leads to ischaemia and promotes a pro-fibrotic environment that is accelerated by the transformation of pericytes to myofibroblasts. The enhanced myofibroblast population is known to result in increased production of extracellular matrix components (ECM), a key factor in the development of interstitial fibrosis (Kida & Duffield 2011, Liu 2011). The initiators of, and signalling mechanisms involved in, pericyte detachment, migration and differentiation are not well defined, although genes regulating proteolytic activity and angiogenesis have been implicated (Schrimpf *et al*. 2012). The recent evidence identifying pericytes as the key cell type in the progression of renal fibrosis has met with some controversy (Zeisberg & Duffield 2010). It is well established that myofibroblasts are important in the pathogenesis of fibrosis, although myofibroblasts are thought to arise from a number of different sources, including differentiation of resident fibroblasts (Liu 2006, 2011, Wada *et al*. 2007). Epithelial cells undergoing epithelial to mesenchymal transition (EMT) were thought to contribute to the myofibroblast pool; however, the occurrence of EMT *in vivo* is still heavily debated (Wada *et al*. 2007, Humphreys *et al*. 2010, Zeisberg & Duffield 2010, Kriz *et al*. 2011, Liu 2011). Recent genetic ‘fate-mapping’ studies indicate that epithelial cells are unable to migrate from the endothelial compartment, and instead, they implicate pericytes as the myofibroblast progenitor (Lin *et al*. 2008, Humphreys *et al*. 2010). Unfortunately, this is difficult to demonstrate *in vivo* or *ex vivo* using cell markers because alpha-SMA, a commonly used myofibroblast marker, is also expressed by pericytes (Park *et al*. 1997, Strutz & Zeisberg 2006). Moreover, the anti-NG2 antibody used to identify pericytes has also been used to identify myofibroblasts in other tissues (Terada *et al*. 2006). The lack of specific markers for pericytes and myofibroblasts has hindered researchers' efforts to clarify the origin of myofibroblasts (Strutz & Zeisberg 2006). More selective markers of these cells, or transgenic animal models, may help to determine the role of pericytes in renal fibrosis.

## Methods for assessing pericyte activity and future directions

A variety of experimental techniques from *in vitro* cell culture approaches to isolated vessel preparations and LDF have been employed to further our understanding of MBF regulation. To make significant advances, the technical problem of renal medulla inaccessibility must first be overcome. As already discussed, early approaches to the investigation of MBF regulation based on the LDF technique or the dual-slit imaging method have been inconclusive. To date, the best-established technique for studying pericyte-mediated regulation of vasa recta is the isolated perfused DVR method (Pallone 1994). Although this model has provided much of our knowledge about the vasoactivity of these cells, it does not allow tubulo-vascular cross-talk mechanisms to be explored. Studies performed on microtissue strips of medulla have alluded to ‘tubular-vascular cross-talk’ in experiments that demonstrated that Ang-II-evoked constriction in isolated vasa recta is buffered by locally produced NO in adjacent tubules (Dickhout *et al*. 2002). This finding is consistent with the hypothesis of tubulo-vascular cross-talk *in vivo*, and it is at least consistent with our proposal that pericytes may be key mediators. The *in vitro* blood-perfused juxtamedullary technique (Casellas & Navar 1984) has also been used extensively to investigate afferent and efferent arteriolar blood flow (Harrison-Bernard & Carmines 1994, 1995, Takenaka *et al*. 1994), and the application of certain elements of this technique may indeed improve current methodologies, that is, perfusion of DVR within slices.

Mathematical modelling has also been applied to investigate the regulation of MBF (Zhang & Edwards 2007). This computational model provides a two-dimensional representation of the inner stripe of the outer medulla and was constructed by randomly distributing a number of tubular and vascular structures within a concentric region. The model is adapted from the configuration initially published by Layton and Layton, which was based on data collated from *in vivo* and *in vitro* experiments (Layton & Layton 2005, Zhang & Edwards 2007). Although the construction of such a model may be subject to ‘investigator manipulation’, it can provide useful insights into the generation and distribution of endogenous vasoactive stimuli.

A recent development of a live kidney slice model provides an alternative *in vitro* experimental model in which medullary structures can be investigated *in situ*. As with most techniques, this model is not without its limitations. For example, it is not possible to simulate the osmotic and oxygen gradients present *in vivo*, and DVR in the live slice model are not perfused (Crawford *et al*. 2012). Perfusion of tubular and/or vascular structures within the tissue slice, to more accurately assess the intraluminal signalling pathways, would offer an obvious (yet technically challenging) improvement. Despite these limitations, this method uniquely allows visualization of pericytes in their *in situ* environment, and data collected using this model have confirmed that *in vitro* observations regarding pericyte activity are reproducible, and new insights into pericyte activity and regulation are being described (Crawford *et al*. 2011, 2012). A significant and noteworthy advantage of this model is that the relationship between pericytes and the surrounding interstitial and tubular cells can be investigated, and this has been key to demonstrating the role of pericytes in tubulo-vascular cross-talk (Crawford *et al*. 2012). Although the live tissue slice model is relatively new in renal blood flow studies, the slice technique *per se* is a well-established model for investigating brain function and has previously been used to investigate the role of pericytes in regulating the cerebellar microcirculation (Peppiatt *et al*. 2006). We expect this experimental model will complement existing approaches employed to investigating renal microvascular function and hope that future investigations using this model will facilitate the delineation of MBF regulatory mechanisms.

In summary, there appears to be significant experimental evidence, which favours regulated blood flow in the medulla, and this complements the need for maintained homoeostasis in this region. We propose pericytes are key players in the regulation of renal medullary function, a proposal that is supported not only by their physical location on the vasa recta, but also by evidence describing their ability to respond to endogenous vasoactive agents originating from neighbouring tubular and interstitial cells. There is still much to understand about renal pericyte activity, particularly about their ability to communicate with neighbouring structures and the way in which they transcribe changes in tubular function to changes in blood flow. However, technical advances coupled with increased interest, particularly because of the implication of pericytes in renal disease, should rapidly lead to increased understanding of pericyte activity in renal physiology and pathophysiology.
